# Residual transpiration as a component of salinity stress tolerance mechanism: a case study for barley

**DOI:** 10.1186/s12870-017-1054-y

**Published:** 2017-06-19

**Authors:** Md. Hasanuzzaman, Noel W. Davies, Lana Shabala, Meixue Zhou, Tim J. Brodribb, Sergey Shabala

**Affiliations:** 1 School of Land and Food, University of Tasmania, Private Bag 54, Hobart, Tas 7001 Australia; 2 Central Science Laboratory, University of Tasmania, Hobart, Tas 7001 Australia; 3 School of Biological Science, University of Tasmania, Private Bag 55, Hobart, Tas 7001 Australia; 4 Department of Agronomy, Faculty of Agriculture, Sher-e-Bangla Agricultural University, Sher-e-Bangla Nagar, Dhaka, -1207 Bangladesh

**Keywords:** Residual transpiration, Osmolality, Osmotic potential, Leaf water potential, Cuticular waxes

## Abstract

**Background:**

While most water loss from leaf surfaces occurs via stomata, part of this loss also occurs through the leaf cuticle, even when the stomata are fully closed. This component, termed residual transpiration, dominates during the night and also becomes critical under stress conditions such as drought or salinity. Reducing residual transpiration might therefore be a potentially useful mechanism for improving plant performance when water availability is reduced (e.g. under saline or drought stress conditions). One way of reducing residual transpiration may be via increased accumulation of waxes on the surface of leaf. Residual transpiration and wax constituents may vary with leaf age and position as well as between genotypes. This study used barley genotypes contrasting in salinity stress tolerance to evaluate the contribution of residual transpiration to the overall salt tolerance, and also investigated what role cuticular waxes play in this process. Leaves of three different positions (old, intermediate and young) were used.

**Results:**

Our results show that residual transpiration was higher in old leaves than the young flag leaves, correlated negatively with the osmolality, and was positively associated with the osmotic and leaf water potentials. Salt tolerant varieties transpired more water than the sensitive variety under normal growth conditions. Cuticular waxes on barley leaves were dominated by primary alcohols (84.7–86.9%) and also included aldehydes (8.90–10.1%), *n-*alkanes (1.31–1.77%), benzoate esters (0.44–0.52%), phytol related compounds (0.22–0.53%), fatty acid methyl esters (0.14–0.33%), β-diketones (0.07–0.23%) and alkylresorcinols (1.65–3.58%). A significant negative correlation was found between residual transpiration and total wax content, and residual transpiration correlated significantly with the amount of primary alcohols.

**Conclusions:**

Both leaf osmolality and the amount of total cuticular wax are involved in controlling cuticular water loss from barley leaves under well irrigated conditions. A significant and negative relationship between the amount of primary alcohols and a residual transpiration implies that some cuticular wax constituents act as a water barrier on plant leaf surface and thus contribute to salinity stress tolerance. It is suggested that residual transpiration could be a fundamental mechanism by which plants optimize water use efficiency under stress conditions.

**Electronic supplementary material:**

The online version of this article (doi:10.1186/s12870-017-1054-y) contains supplementary material, which is available to authorized users.

## Background

Under optimal conditions plants lose typically 95–98% water from the leaf surface via stomatal pores in a process termed stomatal transpiration. However, under some environmental conditions, a relatively large portion of evaporated water may bypass the stomata and occur through the cuticle. Depending on the species and conditions, water loss through the cuticle can be as high as 28% of the water transpired through stomata [1, 2]. Moreover, some water can escape the leaf via stomata even when they are fully closed [3, 4]. Because of this, using the term “cuticular transpiration” is not always appropriate, and this process is best described as “residual transpiration”. It has been estimated that leaf cuticular water permeability varies extensively among species and ranges from 10^−7^ to 10^−4^ m s^−1^ [2, 5]. Residual transpiration is usually localized to the area surrounding stomata, where there are more and larger cuticular pores [6]. While stomatal conductance is a dynamic process that can be rapidly controlled by ion fluxes into/out of guard cells, residual transpiration depends almost entirely on the existing (passive) lipophilic cuticular pathway of the leaf surface, and, hence cannot rapidly be adjusted to changing conditions [7, 8]. However, when stomata are closed under salinity or drought conditions, the balance between stomatal and non-stomatal transpiration is shifted. Under severe stress conditions, when stomata are closed and stomatal transpiration is reduced to nearly zero, the difference in residual transpiration becomes a significant factor determining water use efficiency. Thus, reducing non-stomatal (residual) transpiration is a potentially useful mechanism for improving plant performance under stress conditions. Genotypes having lower residual transpiration can conserve relatively more water under water stress conditions, and it has therefore been suggested as a selection trait in the breeding of cereals genotypes adapted to a dry environment [9, 10].

Cuticular wax is the outermost hydrophobic layer of the aerial plant tissues, and plays an important role in protecting plants against biotic and abiotic environmental stresses, and acts as a barrier to excessive non-stomatal transpiration [11]. The main functions of cuticular waxes include maintaining equilibrium between the transpirational water loss and root water uptake by transpiration control, defending against attack by insects and pathogens, reducing water retention on plant surfaces by controlling surface wettability, controlling loss and uptake of polar solutes, and regulating the exchange of gases and vapour [12]. Extraction of cuticular waxes from plant parts with organic solvent increases the cuticular water permeability indicating that the wax layer is a fundamental water transport-limiting barrier of the cuticle, especially when stomata are closed [13]. Some reports suggested that plants that have a thicker cuticle or a cuticle containing a larger amount of waxes are more efficient in reducing non-stomatal transpiration and thus better adapted to water stress conditions [14], and in some species total wax loads increased by 30 to 70% under water stress conditions [15]. However, the correlation between residual transpiration and the thickness of cuticle and/or amount of total cuticular waxes is still not clear-cut. Some researchers found that the total amount of cuticular waxes and cuticular thickness are negatively correlated with residual transpiration in different plants [16–19]. However, some authors reported no correlation between residual transpiration and waxes [2, 20, 21].

Residual transpiration could be influenced by the characteristics of the leaf surface and morphological structure of the plant. Some studies argued [2] that residual transpiration did not relate to the amount of wax coverage and thickness of the cuticle but could be dependant on physical properties, orientation of wax crystal structure and wax composition. It is not clear however if this conclusion can be extrapolated to all species. The cuticle layer is a cutin-rich domain with embedded polysaccharides and an overlying layer that is less abundant in polysaccharides but enriched in waxes referred to as the cuticle proper [11]. The waxes are either deposited within the cutin matrix known as intracuticular wax or accumulate on its surface known as epicuticular wax crystals, or films. Cuticular waxes is a general term for the complex mixture of homologous series of very-long-chain fatty acids, primary *n-*alcohols, secondary *n-*alcohols, *n-*aldehydes, *n-*alkanes, *n-*alkyl esters, and cyclic organic compounds like pentacyclic triterpenoids, flavonoids, tocopherols and hydroxycinnamic acids derivatives [22]. Specific chemical compounds of the cuticle may be related to the water barrier. Higher levels of nonpolar long chain aliphatic wax compounds of cuticular wax such as hydrophobic alcohols, *n*-alkanes, and aldehydes tend to be associated with a barrier against cuticular water loss while alicyclic wax components including triterpenoids and sterol derivatives are less effective as a water barrier [23–26].

It is also not clear whether residual transpiration is only related to the cuticular wax on the leaf surface or it is also associated with the plant water relations. It was suggested that residual transpiration is correlated with leaf water status such as leaf water content, osmotic potential and leaf water potential [9]. Other evidence however shown that residual transpiration is not related to relative water content or osmotic potential [27].

The objectives of this study were to investigate the effect of residual transpiration on salinity tolerance and the relationship of residual transpiration to plant water relations, and cuticular wax load at three different leaf positions under irrigated conditions of two salt tolerant and two salt sensitive barley genotypes.

## Methods

### Plant materials and growth conditions

Four barley (*Hordeum vulgare* L.) genotypes contrasting in their salt tolerance were used in this study. Cultivars Franklin and Gairdner were salt sensitive and failed to produce any grain when grown under highly saline (300 mM NaCl) conditions in the glasshouse [28], while cultivars TX9425 and ZUG293 were salt tolerant and managed to produce ~30% increased grain yield (compared with control) under same conditions. Seeds were obtained from the Australian Winter Cereal Collection and multiplied in the field at Tasmanian Institute of Agriculture facilities in Launceston. Seeds were surface sterilized with 10% commercial bleach and thoroughly rinsed with tap water, and sown in 2 L plastic pots using standard potting mixture containing 70% composted pine bark; 20% coarse sand; 10% sphagnum peat; Limil at 1.8 kg m^−3^, dolomite at 1.8 kg m^−3^. The plant nutrient balance was maintained by adding the slow release Osmocote Plus™ fertilizer (at 6 kg m^−3^), plus ferrous sulphate (at 500 g m^−3^). Plants were grown under controlled glasshouse condition (day length 14 h; day/night temperatures 25/15 °C; relative humidity 65%) at the University of Tasmania (Hobart, Australia) in January 2015. The plants were irrigated automatically twice per day.

### Residual transpiration measurement

Two different methods were used for the determination of residual transpiration from the excised leaf under dark conditions as follows:

- *Method-1*

**Fig. 1:**
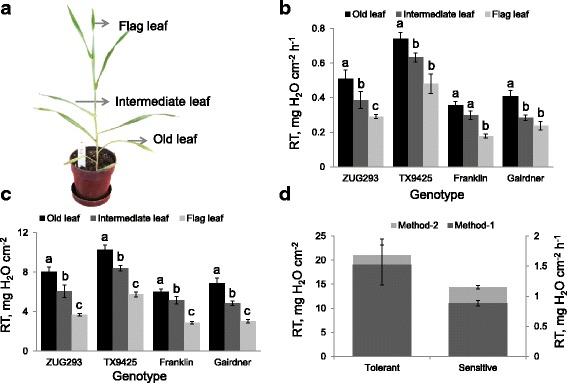
Quantifying the residual transpiration (RT) from leaves of three different positions in barley. **a** sampled leaves; **b**, **c** RT values measured from leaves of three different positions from 4 barley varieties contrasting in salinity stress tolerance by Method-1 and Method-2, respectively. Data is mean ± SE (*n* = 6). **d** mean RT values for plants in salt-tolerant (ZUG293, TX9425) and salt-sensitive (Gairdner, Franklin) groups estimated by two different methods. Data labelled with different *lower case letters* in panels (**b**) and (**c**) are significantly different at *P* < 0.05

Residual transpiration was determined following Clarke and McCaig [29] with modification. Three fully expanded leaves from each genotype at three positions (old leaf, intermediate leaf and young flag leaf) were selected for sampling (Fig. 1a). The leaves were excised and sealed with vacuum grease on the cut end immediately. Then collected leaves were immediately transported to the laboratory. Fresh weights (W_0_) were determined by an electronic balance. The leaves were then placed in a controlled dark room at 20–21 °C and 50% relative humidity (RH). The leaves were weighed at 2, 4 and 6 h (W_2_, W_4_ and W_6_ respectively) intervals and then placed in dry oven at 60 °C for 24 h and reweighed (W_d_). Residual transpiration was measured per dry weight basis by using the following formula

$$ \mathrm{Residual}\ \mathrm{transpiration}=\frac{\left({\mathrm{W}}_0-{\mathrm{W}}_2\right)+\left({\mathrm{W}}_2-{\mathrm{W}}_4\right)+\left({\mathrm{W}}_4-{\mathrm{W}}_6\right)}{3\times {\mathrm{W}}_{\mathrm{d}}\left({\mathrm{T}}_2-{\mathrm{T}}_1\right)} $$

where T_1_-T_2_ = time interval between two subsequent measurements (2 h).

The measured residual transpiration was then recalculated per projected leaf area basis and expressed in mg H_2_O cm^−2^ h^−1^.

- *Method-2*

Residual transpiration was measured according to Clarke and co-authors [9] with modification. Leaf sampling was the same as for Method-1. Initial weights were determined immediately after excision of leaves. The leaves were maintained in darkness for stomatal closure under ambient room conditions at 20–21 °C and 50% RH. The leaves were weighed again after 24 h. The leaves were dried at 60 °C for 24 h and then dry weight was determined. Residual water loss was determined per dry weight basis by using the following formula

$$ \mathrm{Residual}\ \mathrm{transpiration}=\frac{\left({\mathrm{W}}_{\mathrm{i}}-{\mathrm{W}}_{\mathrm{d}}\right)-\left({\mathrm{W}}_{24}-{\mathrm{W}}_{\mathrm{d}}\right)}{{\mathrm{W}}_{\mathrm{d}}} $$

where W_i_ = Initial fresh weight; W_24_ = Fresh weight after 24 h; W_d_ = Dry weight

The measured residual water loss was then recalculated per leaf area basis and expressed in mg H_2_O cm^−2^.

### Measurement of leaf osmolality and osmotic potential

Three leaves at three leaf position e.g. old, intermediate and young flag leaves were taken from each genotype. Representative leaf samples were taken in centrifuge tubes and frozen at −20 °C overnight and then squeezed to extract sap. An amount of 10 μl sap was taken from each sample for measuring leaf osmolality (c) using a vapour pressure osmometer (Vapro model 5520, Wescor Inc., Logan, Utah). The osmotic potential was calculated by Van’t Hoff’s equation from the osmolality (mmol kg^−1^): osmotic potential (MPa) = −c (mmol kg^−1^) × 2.4789 × 10^−3^ at 25 °C.

### Measurement of leaf water potential

Two leaves were excised from each genotype from three positions of the stem for leaf water potential determinations. The leaf blades were cut with a sharp blade and immediately sealed in an elliptical grass compression gland gasket. The leaf blades were sealed in a pressure chamber (Model 615; PMS Instruments, Albany, OR, USA), and the chamber was pressurized using compressed air at a rate of 0.1 MPa s^−1^ until water first appeared at the cut surface of the leaf. The total elapsed time from when the leaf was cut from the plant to the initial pressurisation of the chamber was 5–10 s. The leaf water potential data were reported in MPa.

### Scanning electron microscopy (SEM)

After sampling the leaves were stored at −20 °C overnight and then lyophylised in a pre-cooled freeze drier (Mini-ultra cold, Dynavac, Aus, Techno lab). The dried samples (3–5 mm long) were mounted on SEM specimen stubs with double-sided carbon tape (one half with adaxial and the other with abaxial surface uppermost) and then coated with a thin film (2–3 nm) of Pt for 20 min using a sputter coater (BalTec SCD 050) in an atmosphere of argon to improve the electrically conducting properties of leaf and high resolution of images. Three replicates of coated samples were examined with a Hitachi SU-70 UHR field emission scanning electron microscope setting with 1.5 kV, 17.2 mm × 2.00 k SE (M). The imaging was performed in the Central Science Laboratory, University of Tasmania.

### Wax extraction and analysis

Three fresh leaves at three positions of the plant from each genotype were excised and ten 0.64 cm^2^ disks were sampled from each by leaf punch.. The leaf segments were soaked in 5 mL of solvent (dichloromethane with *n*-docosane (C_22_ alkane, 20 mg/L) as an internal standard) for 5 min with gentle stirring [30]. The extract contained waxes from both abaxial and adaxial leaf surfaces. The extracts were evaporated to dryness under a nitrogen stream for 30 min at 58 °C. The samples were redissolved in 0.5 ml dichloromethane for analysis by combined gas chromatography-mass spectrometry (GC-MS) on a Varian 3800 gas chromatograph coupled to a Bruker-300 triple quadrupole mass spectrometer. One microlitre injections in splitless mode were made with an injector temperature of 275 °C. The column was a 30 m × 0.25 mm DB5 (0.25 μm film thickness) (Agilent, Australia) and the oven temperature program was 60 °C (held for 1 min) to 220 °C at 30 °C per minute, then to 310 °C at 10 °C per minute with a final hold time of 5 min. The carrier gas was helium at a constant flow of 3.5 ml min^−1^. Mass spectra were collected over the range *m/z* 40 to 600 every 0.3 s. Compounds were identified through a combination of MS reference databases (NIST MS database and an in-house database of relevant compounds), and Kovats’ retention indices. The individual components and total wax were expressed in terms of μg equivalents of *n-*docosane cm^−2^. All subsequent μg cm^−2^ values are in terms of *n-*docosane equivalents in the text and figures.

### Statistical analysis

All data were analyzed by using SPSS 20.0 for Windows (SPSS Inc.). Significant differences between different genotypes were determined by one-way analysis of variance based on Duncan’s multiple range tests. Different lower case letters in the figures represent significant differences. The significance of correlations between different parameters was determined by bivariate correlations based on Pearson’s correlation (two-tailed).

## Results

### Residual transpiration

As both stomata density and amount of cuticular waxes depends on the leaf age, we hypothesised that a significant variation in residual transpiration should exist between leaves of different positions. A significant variation was seen in the different leaf positions for all varieties (*P* < 0.05; Fig. 1a and b). Old leaves transpired more water than the intermediate and flag leaves for all varieties using both methods. In Method-1, significant variation was observed between old leaves and intermediate leaves but not in intermediate and flag leaves in most genotypes. Old leaves of TX9425 (0.74 ± 0.04 mg H_2_O cm^−2^ h^−1^) genotype transpired the highest amount of water and Franklin transpired the lowest amount of water (0.36 ± 0.02 mg H_2_O cm^−2^ h^−1^). In Method-2, significant differences were seen between the three leaf position in all genotypes. Old leaves of TX9425 (10.24 ± 0.53 mg H_2_O cm^−2^) transpired the highest amount of water followed by old leaves of ZUG293 (8.01 ± 0.48 mg H_2_O cm^−2^), Gairdner (6.88 ± 0.52 mg H_2_O cm^−2^) and Franklin (6.02 ± 0.28 mg H_2_O cm^−2^), respectively. Young flag leaves of TX9425 (5.73 ± 0.25 mg H_2_O cm^−2^) transpired the highest amount of water followed by ZUG293 (3.68 ± 0.14 mg H_2_O cm^−2^), Gairdner (3.02 ± 0.17 mg H_2_O cm^−2^) and Franklin (2.86 ± 0.12 mg H_2_O cm^−2^), respectively. Salt tolerant varieties transpired more water through the cuticle than that of sensitive varieties under normal growth conditions (Fig. 1c). The cumulative loss of water of the three leaf positions of two tolerant genotypes (TX9425 and ZUG293) was higher than two sensitive genotypes (Gairdner and Franklin) in both methods. The two tolerant genotypes transpired 43% and 32% more water respectively than the two sensitive genotypes in the two methods under normal growth condition.

### Leaf sap osmolality correlates negatively with residual transpiration

**Fig. 2:**
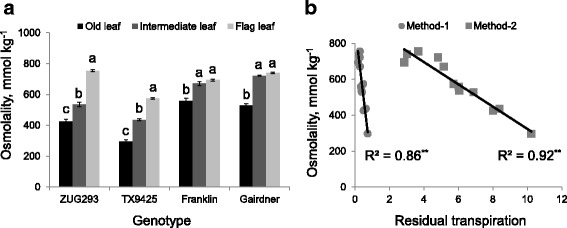
**a** genetic variability in osmolality of barley leaves at three positions in plants grown under normal (no salt) growth condition. Mean ± SE (*n* = 6). **b** correlations (Pearson’s R^2^ values) between leaf sap osmolality and residual transpiration measured by two different methods. Data labelled with different *lower case letters* are significantly different at *P* < 0.05 and *asterisks* are significant at *P* < 0.01

**Fig. 3:**
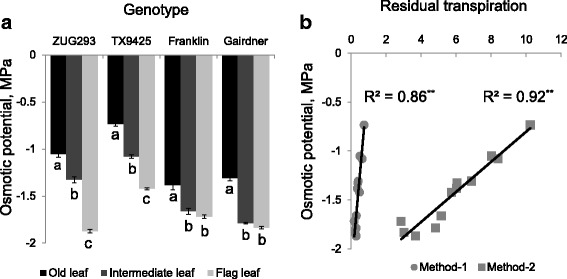
**a** genetic variability in osmotic potential of barley leaves at three positions in plants grown under normal (no salt) condition. Mean ± SE (*n* = 6). **b** correlations (Pearson’s R^2^ values) between leaf osmotic potential and residual transpiration measured by two different methods. Data labelled with different *lower case letters* are significantly different at *P* < 0.05 and *asterisks* are significant at *P* < 0.01

A significant difference of leaf sap osmolality was observed among different leaf positions (*P* < 0.05; Fig. 2a). Leaf sap osmolality decreased with increasing leaf age for all genotypes. The osmotic potential was highest in old leaf and lowest in flag leaf in all genotypes (*P* < 0.05; Fig. 3a). The highest decrease (60%) was observed in TX9425 followed by ZUG293 (43%), whereas the lowest decrease (20%) was measured in Franklin followed by Gairdner (28%), in old and young leaves respectively. A strong negative correlation (R^2^ = −0.86 for Method-1 and -0.92 for Method-2; significant at *P* < 0.01) was found between the overall leaf sap osmolality in plants grown under normal growth conditions and residual transpiration.

### Osmotic potential and leaf water potential correlate positively with residual transpiration

**Fig. 4:**
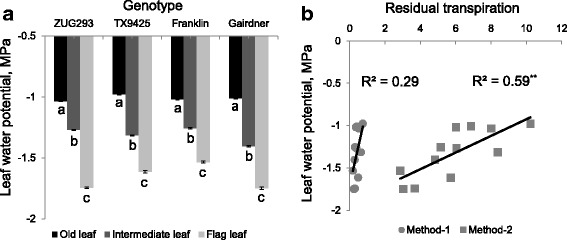
**a** genetic variability in water potential of barley leaves at three positions in plants grown under normal (no salt) growth condition. Mean ± SE (*n* = 6). **b** correlations (Pearson’s R^2^ values) between leaf water potential and residual transpiration measured by two different methods. Data labelled with different *lower case letters* are significantly different at *P* < 0.05 and *asterisks* are significant at *P* < 0.05

The osmotic potential was highest in old leaves and lowest in flag leaves in all genotypes (*P* < 0.05; Fig. 3a). ZUG293 and TX9425 followed the order old > intermediate > young flag leaf, whereas Franklin and Gairdner followed old > intermediate = young flag leaf. A strong positive correlation (R^2^ = 0.86 for Method-1 and 0.92 for Method-2; significant at *P* < 0.01) was found between the overall leaf osmotic potential in plants grown under normal growth conditions and residual transpiration. A significant variation of leaf water potential was found among the three leaf positions in all four genotypes (*P* < 0.05; Fig. 4a). Leaf water potential increased with increasing the plant leaf age, the highest and lowest leaf water potential was found at old leaf and young flag leaf, respectively. A positive correlation (R^2^ = 0.59; significant at *P* < 0.01) was found (in Method 2) between the overall leaf water potential in plants grown under normal growth condition and residual transpiration.

### Structure and distribution of cuticular waxes on leaf epidermis

**Fig. 5:**
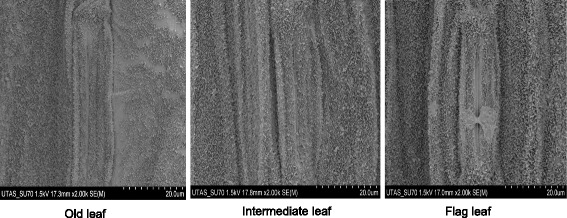
Representative SEM images showing cuticular wax on the adaxial surface in leaves of three different positions in variety Franklin grown under control condition. One (of six) typical images is shown for each position

**Fig. 6:**
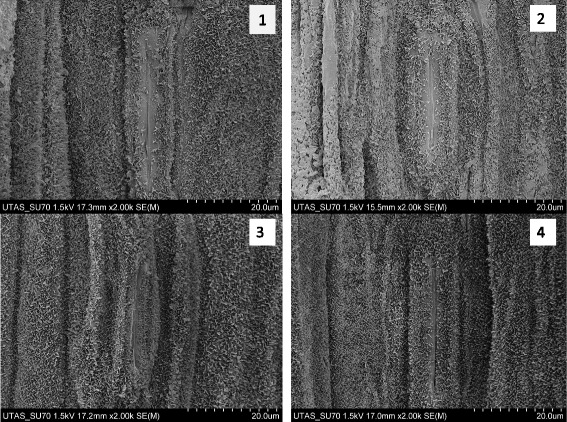
Representative SEM images showing cuticular wax on the adaxial surface of the flag leaf in barley varieties ZUG293 (**1**), TX9425 (**2**), Franklin (**3**) and Gairdner (**4**) grown under control conditions. One (of six) typical images is shown for each genotype

SEM analysis showed similar cuticular waxes structure in three different leaf positions of four barley genotypes. The cuticular waxes formed combined coatings of different arrangement of minute crystallised plates about 1–2 μm in size, relatively vertically oriented to the leaf epidermal surface (Fig. 5; Additional file 1: Figure S1). Cuticular wax structures were a less dense covering of adaxial surface of old leaves compared to the intermediate and young flag leaves for all genotypes. The epidermis of three different leaf positions of four genotypes was covered with waxy plates, but not fully over the guard cell of all genotypes (Fig. 6). In the case of TX9425 and ZUG293 genotypes, the guard cells of stomata were not fully covered with waxy plates, whereas the guard cells of Franklin and Gairdner were fully covered with waxy plates. No differences were found for adaxial and abaxial surface of leaves in all genotypes regarding to cuticular wax structure and density (data not shown).

### Total wax content of leaves correlates negatively with residual transpiration

**Fig. 7:**
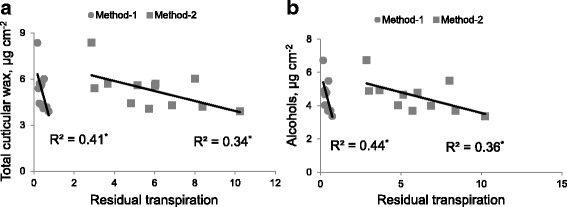
**a** correlations (Pearson’s R^2^ values) between total cuticular wax and residual transpiration measured by Method-1 (mg H_2_O cm^−2^ h^−1^) and Method-2 (mg H_2_O cm^−2^). **b** correlations (Pearson’s R^2^ values) between alcohols and residual transpiration measured by Method-1 (mg H_2_O cm^−2^ h^−1^) and Method-2 (mg H_2_O cm^−2^). Data labelled with *asterisks* are significant at *P* < 0.05

A significant negative correlation (R^2^ = −0.41 for Method-1 and -0.34 for Method-2; significant at *P* < 0.05) was found between the total cuticular wax content of leaves and residual transpiration measured by two different methods in plants grown under normal growth conditions (Fig. 7a).

### Cuticular wax constituents, contents and effect on residual transpiration

Across all four barley varieties the average of total leaf cuticular wax was found to be 5.37 μg cm^−2^ under normal growth condition. The averages of total cuticular wax of old leaves, intermediate leaves and flag leaves of all genotypes studied were 5.06 μg cm^−2^, 5.06 μg cm^−2^ and 5.98 μg cm^−2^ respectively. Cuticular waxes on barley leaves were dominated by primary alcohols (84.7–86.9%), aldehydes (8.90–10.1%), *n-*alkanes (1.31–1.77%), benzoate esters (0.44–0.52%), a phytol related compound (0.22–0.53%), fatty acid methyl esters (0.14–0.33%), β-diketones (0.07–0.23%) and alkylresorcinols constituents (1.65–3.58%). Primary alcohols consisted of odd and even numbers of carbon from C_22_ to C_29_, particularly *n-*docosanol (C_22_), *n-*tetracosanol (C_24_), *n-*hexacosanol (C_26_), and *n-*octasonanol (C_28_), and much smaller amount of odd numbered carbons. The higher *n*-alkane component on barley leaf consisted mainly of *n-*hentriacontane (C_31_) and *n-*tritriacontane (C_33_). The main aldehydes were *n-*hexacosanal (C_26_), *n-*octacosanal (C_28_) and *n-*triacontanal (C_30_). Benzoate esters included *n-*docosyl benzoate (C_22_), *n-*tetracosyl benzoate (C_24_) and *n-*hexacosyl benzoate (C_26_). Major fatty acid methyl esters were methyl *n-*octacosanoate (C_28_), methyl *n-*triacontanoate (C_30_) and methyl *n-*dotriacontanoate (C_32_).

Old leaves for all genotypes studied showed the average highest absolute amount of alcohols (4.39 μg cm^−2^) followed by aldehydes (0.45 μg cm^−2^) and the lowest β-diketones (Table 1). Similar results were found at intermediate and flag leaves for all genotypes (Tables 2 and 3; Additional file 2: Table S1). Among the genotypes, ZUG293 old leaves contained the highest amount of alcohols followed by Franklin. The same results were found for intermediate leaf for all genotypes (Table 2; Additional file 2: Table S1). For flag leaves of all genotypes the average highest alcohols were measured from Franklin followed by ZUG293 (Table 3).

**Table 1:** Absolute amount (μg cm^−2^) of different compounds of cuticular wax on old leaf position of four barley genotypes grown under normal growth conditions (*n* = 4)

Compound	Genotype				
	ZUG293	TX9425	Franklin	Gairdner	Average
Alcohols	5.48 ± 0.16	3.35 ± 0.65	4.76 ± 0.55	3.99 ± 0.27	4.40
Aldehydes	0.38 ± 0.06	0.46 ± 0.09	0.64 ± 0.06	0.32 ± 0.01	0.45
Alkanes	0.07 ± 0.01	0.08 ± 0.01	0.07 ± 0.01	0.05 ± 0.00	0.07
Benzoate esters	0.02 ± 0.00	0.03 ± 0.01	0.03 ± 0.00	0.01 ± 0.00	0.02
Phytol related	0.01 ± 0.00	0.03 ± 0.01	0.04 ± 0.00	0.02 ± 0.00	0.03
Methyl esters	0.03 ± 0.01	0.00 ± 0.00	0.00 ± 0.00	0.00 ± 0.00	0.01
Diketones	0.01 ± 0.00	0.02 ± 0.01	0.00 ± 0.00	0.00 ± 00	0.01
Alkylresorcinols	0.15 ± 0.05	0.00 ± .00	0.15 ± 0.06	0.04 ± 0.01	0.09

**Table 2:** Absolute amount (μg cm^−2^) of different compounds of cuticular wax on intermediate leaf position of four barley genotypes grown under normal growth conditions (*n* = 4)

Compound	Genotype				
	ZUG293	TX9425	Franklin	Gairdner	Average
Alcohols	4.78 ± 0.08	3.69 ± 0.44	4.65 ± 0.29	4.02 ± 0.32	4.29
Aldehydes	0.41 ± 0.02	0.45 ± 0.06	0.65 ± 0.07	0.38 ± 0.03	0.47
Alkanes	0.06 ± 0.01	0.06 ± 0.00	0.07 ± 0.01	0.05 ± 0.00	0.06
Benzoate esters	0.03 ± 0.00	0.03 ± 0.01	0.04 ± 0.01	0.02 ± 0.00	0.03
Phytol related	0.04 ± 0.01	0.01 ± 0.00	0.03 ± 0.00	0.01 ± 0.00	0.02
Methyl esters	0.00 ± 0.00	0.01 ± 0.00	0.00 ± 0.00	0.02 ± 0.00	0.01
Diketones	0.01 ± 0.00	0.01 ± 0.00	0.00 ± 0.00	0.00 ± 0.00	0.01
Alkylresorcinols	0.45 ± 0.01	0.03 ± 0.01	0.21 ± 0.06	0.04 ± 0.01	0.18

**Table 3:** Absolute amount (μg cm^−2^) of different compounds of cuticular wax on flag leaf position of four barley genotypes grown under normal growth condition (*n* = 4)

Compound	Genotype				
	ZUG293	TX9425	Franklin	Gairdner	Average
Alcohols	4.93 ± 0.21	3.68 ± 0.41	6.71 ± 0.41	4.88 ± 0.17	5.05
Aldehydes	0.40 ± 0.02	0.32 ± 0.05	1.26 ± 0.12	0.45 ± 0.03	0.61
Alkanes	0.06 ± 0.01	0.06 ± 0.00	0.11 ± 0.01	0.06 ± 0.00	0.07
Benzoate esters	0.03 ± 0.00	0.02 ± 0.01	0.05 ± 0.00	0.02 ± 0.00	0.03
Phytol related	0.02 ± 0.00	0.01 ± 0.00	0.02 ± 0.00	0.01 ± 0.00	0.02
Methyl esters	0.01 ± 0.00	0.02 ± 0.00	0.00 ± 0.00	0.05 ± 0.00	0.02
Diketones	0.01 ± 0.00	0.02 ± 0.00	0.01 ± 0.00	0.02 ± 0.00	0.02
Alkylresorcinols	0.36 ± 0.01	0.03 ± 0.00	0.28 ± .00	0.04 ± 0.00	0.18

A negative significant correlation (R^2^ = −0.44 for Method-1; *P* < 0.05 and R^2^ = −0.36 for Method-2; significant at *P* < 0.05) was found between residual transpiration and primary alcohols of cuticular wax component of barley genotypes (Fig. 7b). No significant correlations were found between residual transpiration measured by two different methods and other cuticular wax components (Table 4).

**Table 4:** Correlations (Pearson’s R^2^ values) between residual transpiration measured by two different methods and different cuticular wax compounds of three different leaf positions of four barley genotypes grown under normal growth condition. Values labelled with asterisk are significant at *P* < 0.05

Compound	R^2^ values with residual transpiration				Correlation
	Method-1		Method-2		
	R^2^ Value	*P* value	R^2^ value	*P* value	
Aldehydes	0.21	0.15	0.17	0.18	Negative
Alkanes	0.00	0.93	0.00	0.88	Negative
Benzoates	0.16	0.21	0.15	0.21	Negative
Phytols	0.00	0.86	0.05	0.50	Positive
Methyl esters	0.02	0.63	0.06	0.43	Negative
Diketones	0.04	0.52	0.00	0.89	Positive
Alkylresorcinols	0.21	0.15	0.16	0.19	Negative

## Discussion

### Residual transpiration and plant water relations

To maintain proper growth and leaf expansion, the growing shoot needs to maintain positive turgor which can be achieved by maintaining osmotic cellular adjustment by either increasing the production of compatible solutes or inorganic ions. As plants accumulate more organic osmolytes in young leaves than old leaves to maintain turgor pressure [31], it was hypothesised that residual transpiration should be less in young leaves due to the fact that they have higher osmolality and hence better water retention, and this was found to be the case. As shown in Fig. 2a and b, young flag leaves had a higher osmolality than the older leaves, and increased osmolality had a strong negative correlation with the residual transpiration under normal growth conditions indicating that the increase of leaf sap osmolality might decrease the water transpiration through plant cuticle. An effective osmotic adjustment mechanism may maintain water status in the leaf tissue by decreasing in the cell sap osmotic potential resulting from a net increase of intracellular solutes [32].

A leaf can increase its resistance to dehydration through a reduction in cellular osmotic potential by a net accumulation of cellular solutes. In this study, young flag leaves possessed significantly lower osmotic potential than the intermediate and older leaves; a trend that was correlated positively with residual transpiration (Fig. 3a and b). This indicated that a leaf with lower osmotic potential had more turgor pressure to spend and could resist greater loss of water through the cuticle. Lower negative leaf water potential was measured with increasing leaf age for all varieties, which was negatively correlated with residual transpiration (Fig. 4a and b). Young leaves maintained less turgor at more negative leaf water potentials and tended to have less residual transpiration. Increased turgor in the epidermis stretches cuticles and causes a change in gas exchange of the cuticle. A leaf with less turgor would have a tighter cuticle, thus inhibiting gas exchange [33]. Burghardt and Riederer [14] observed that cuticle gas exchange was affected when leaf water potentials decreased. Thus, leaf water potential affects the diffusion of water vapour through the cuticular barrier, and residual transpiration is negatively correlated with lower leaf water potential [33].

### Change in residual transpiration to improve water use efficiency

Salinity stress is often referred to as a "physiological drought", so some correlation between salinity and drought stress tolerance is expected. The most salinity tolerant varieties showed the highest residual transpiration under unstressed conditions (Fig. 1d). Being somewhat counterintuitive, this is in a good agreement with Bengston et al. [34] who showed that drought stress resistant oat genotypes generally transpired the highest amount of water through the cuticle under unstressed conditions, whereas it was strongly reduced under stress conditions. In addition, higher (33 to 38%) residual transpiration in wheat and cotton leaves was reported from irrigated than rainfed field-grown wheat plants [9]. On the other hand, deposition of cuticular waxes increased in tolerant genotypes during prolonged drought stress, leading to a reduced rate of residual transpiration [16, 35].

Water use efficiency can be expressed as the ratio of leaf net carbon assimilation to total transportation water loss. Plants exhibit higher water use efficiency with higher CO_2_ assimilation than the stomatal conductance, when non-stomatal water loss is negligible [36]. Salt tolerant genotypes transpired more water through cuticle under well irrigated condition that reveals their water use efficiency is lower than sensitive genotypes. Generally stress tolerant barley genotypes have a lower biomass and yield performance under control conditions [37]. This could be due to their higher non-stomatal transpiration under irrigated conditions resulting in lower water use efficiency. Conversely, tolerant genotypes could reduce residual water loss under water deficit conditions when stomata are closed and/or partially closed, and this increased water use efficiency could be a significant factor determining their survival capacity to hostile environmental conditions compared to the standard cultivated genotypes. It has been documented that wheat genotypes having lower residual transpiration adapted and performed better under water stress conditions [38]. Genotypes with normally low residual transpiration are at a functional advantage in water-limited environments since they make more efficient use of the water available. Thus, under conditions of water deficit, residual conductance to water vapour may be an important determinant of plant water balance and stress reactivity.

On the other hand, transpiration is the most effective way of leaf cooling of well-irrigated plants. In plants with adequate water supply stomata may regulate leaf temperature close to the optimum for metabolic processes, including photosynthesis or to prevent tissue heat damage under excessive radiation or temperature [39]. Moreover, under water limited conditions, stomatal closure and decreased transpiration, associated with high water use efficiency, may lead to a dramatic increase in leaf temperature (up to 7 °C above air temperature) [40]. At this condition, high temperatures may disrupt the photosynthetic-related enzymes and produce reactive oxygen species which would challenge the plant cell [41].

### Relationship between residual transpiration and amount of cuticular waxes

Our working hypothesis in this study was that reduced residual transpiration should be positively correlated with hydrophobicity of the leaf surface (hence, amount of cuticular waxes deposited). A significant negative correlation (Fig. 7a) between the total amount of cuticular wax and residual transpiration was found in the present investigation, which indicated that amount of cuticular wax may create a protecting barrier to reduce the loss of water through the cuticle. Previous studies have reported a weak but significant negative correlation between the cuticular wax and residual transpiration in sorghum [18], wheat [17], and barley [42]. This weak correlation may be due to the protecting barrier to the diffusion of water through the cuticle depends on the structure, orientation of wax plates on epidermis, variation of epicuticular and intracuticular wax compositions and distribution of wax plates. Both intracuticular [43] and epicuticular [44] wax layer may contribute to the formation of residual transpiration barrier depending on the plant species and specific cuticle constituents. Plants generally exhibited a significant increase in the amount of cuticular wax amount per unit area of leaves under different stress condition such as water deficit and salinity [20]. The quantity of cuticular wax, however, is not the sole contributor to residual transpiration due to the complexity of water flow through the cuticle [45].

Cuticular waxes have different types of structural morphology including granules, filaments, plates and tubes [12]. According to the SEM images analysis, plate type cuticular wax observed on the leaf surface consisted of aliphatic compounds in which the primary alcohols *n-*hexacosanol and *n-*octacosanol were predominant in different leaf positions for all the barley genotypes.

Cuticular waxes on barley leaves consisted of alcohols, aldehydes, alkanes, benzoate esters, phytol related compounds, fatty acid methyl esters, β-diketones and alkylresorcinols (Tables 1, 2 and 3). Generally, plate type primary alcohol based cuticular waxes always dominate on the leaf surface in the Fabaceae and Poaceae (wheat, barley) [42] and constitute the major barrier to water loss. This was also the case in our study reported here (Fig. 7b) [45]. However, such findings could be not generalized to all species. The hydrophobic long chain alcohol, hydrocarbon and aldehyde fractions are the active components of cuticle in controlling residual transpiration in different plant species [44]. The main portion of the transpiration barrier in tomato fruits and *Rhazya stricta* leaves is located in the intracuticular wax layer containing large amount of pentacyclic triterpenoids whereas cuticular very long chain aliphatics play a minor role [46, 47]. Plant species containing fatty acid with very long aliphatic chain (alcohols, aldehydes and alkanes) in the epicuticular wax, together with high amount of alicyclic compounds such as triterpenoids, steroids, or tocopherols in the intracuticular wax contribute equally to the formation of residual transpiration barrier (44). In general, it is accepted that higher levels of long chain aliphatic components in the wax can lead to a higher hydrophobicity of the residual transpiration barrier and thus decrease cuticular water loss [26]. This should be kept in mind while targeting this trait in the breeding programs.

## Conclusions

Both leaf osmotic potential and the amount of cuticular waxes are involved in controlling water loss from barley leaves under well irrigated conditions. A significant and negative relationship between the amount of primary alcohols and cuticular transpiration implies that primary alcohols may influence the water barrier more than other constituents on plant leaf surface and thus contribute to salinity stress tolerance, at least in barley.

## Additional files

Additional file 1: Figure S1. SEM images showing cuticular wax on the adaxial surface in three different positions of leaf in varieties ZUG293 (A), TX9425 (B) and Gairdner (C) grown under control conditions (PPTX 4472 kb).

Additional file 2: Table S1. Amount (μg cm^−2^) of different components of cuticular wax in three different positions of leaf of four barley genotypes (*n* = 4) (XLSX 12 kb).

## Abbreviations

MS: Mass spectrometry
RH: Relative humidity
RT: Residual transpiration
SEM: Scanning electron microscopy
