# Supplementary material for: Residual transpiration as a component of salinity stress tolerance mechanism: a case study for barley
Source: BMC Plant Biol. 2017 Jun 19;17:107. doi: 10.1186/s12870-017-1054-y (PMC5477354; doi:10.1186/s12870-017-1054-y)
Supplement: Supplementary file 1 — SEM images showing cuticular wax on the adaxial surface in three different positions of leaf in varieties ZUG293 (A), TX9425 (B) and Gairdner (C) grown under control conditions (PPTX 4472 kb). [file 12870_2017_1054_MOESM1_ESM.pptx]

## Slide 1
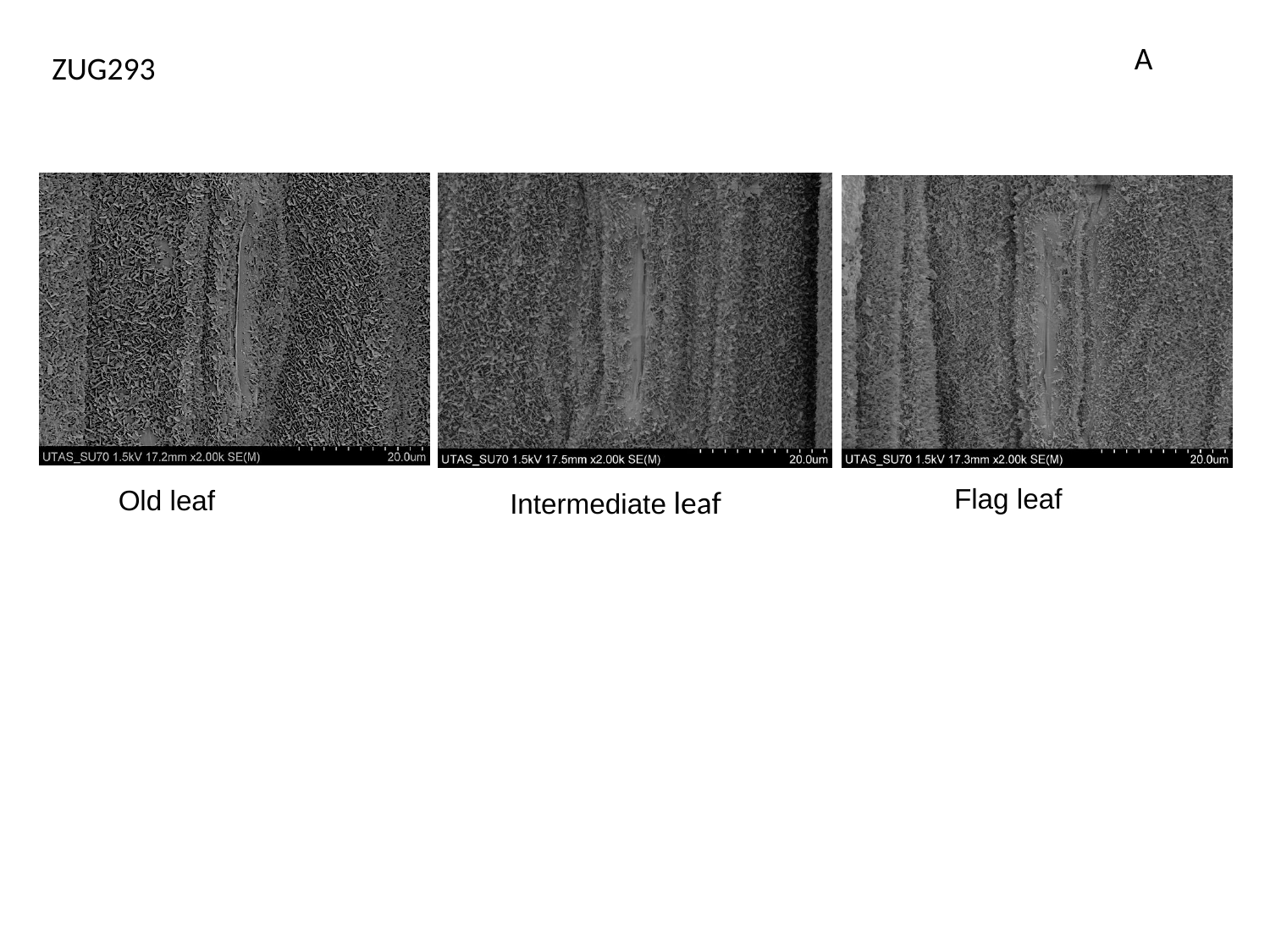

A
ZUG293
Flag leaf
Old leaf
Intermediate leaf

## Slide 2
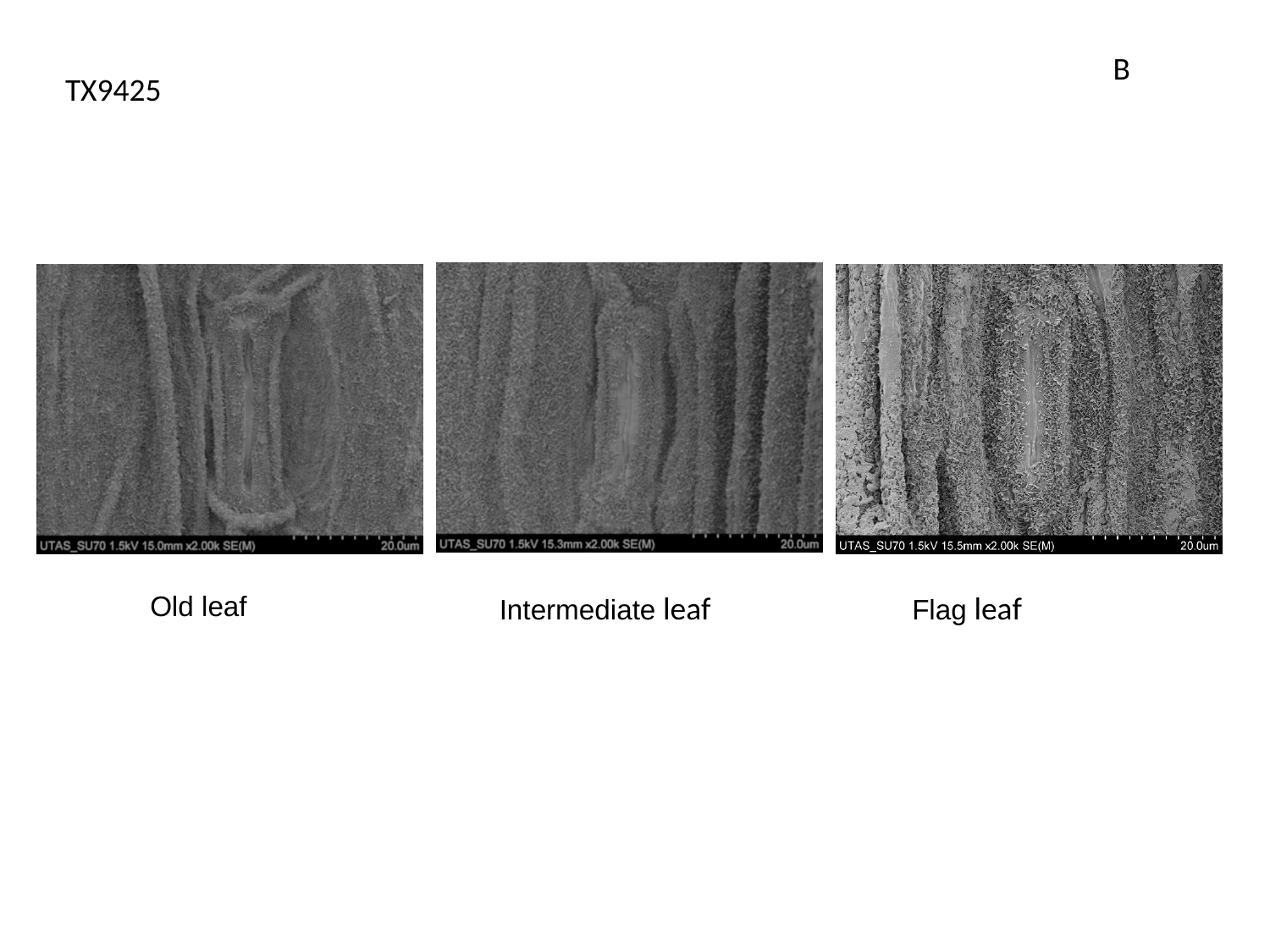

B
TX9425
Old leaf
Intermediate leaf
Flag leaf

## Slide 3
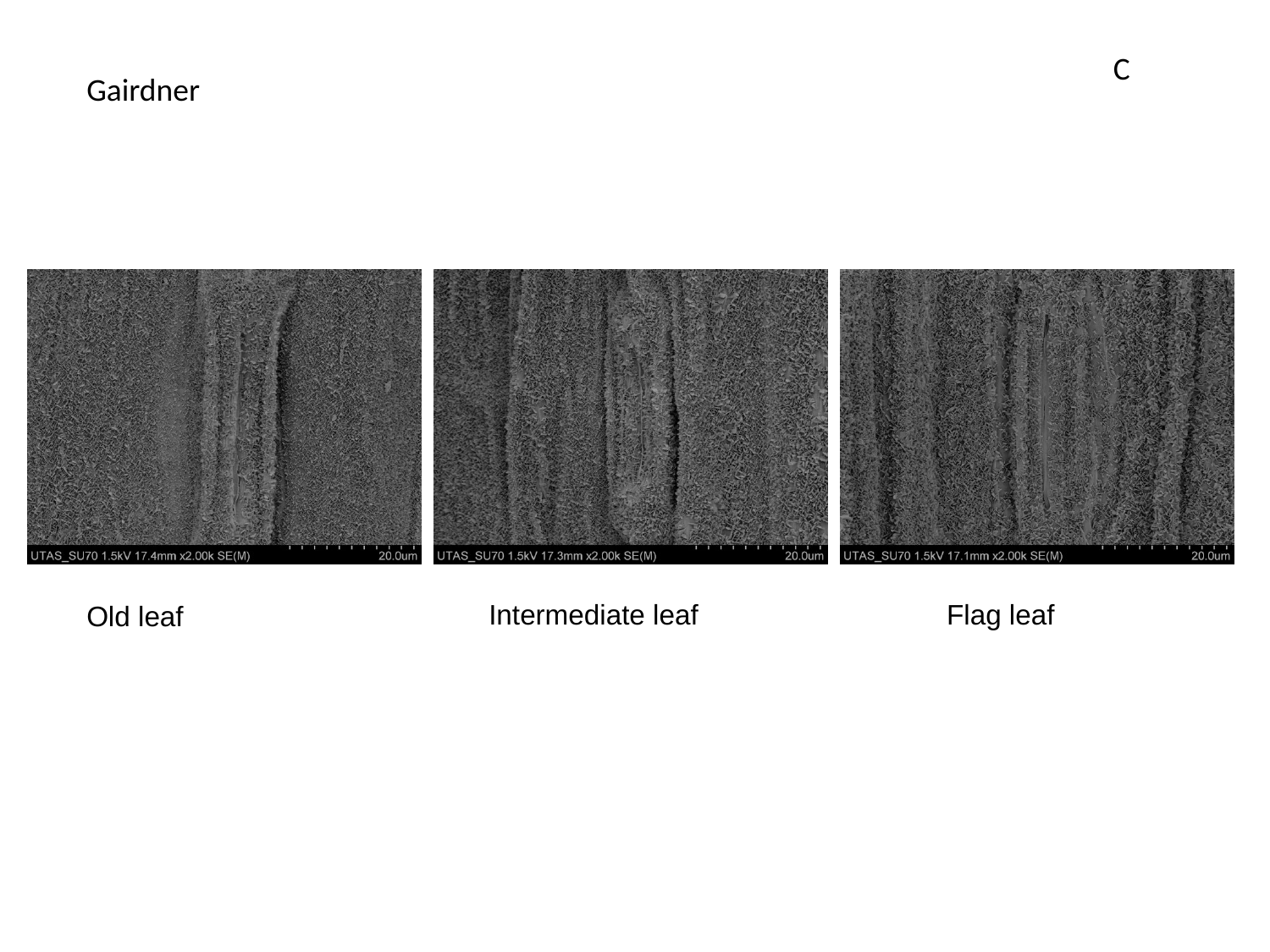

C
Gairdner
Intermediate leaf
Flag leaf
Old leaf
